# High Internal Phase *Oil-in-Water* Emulsions Stabilised by Cost-Effective Rhamnolipid/Alginate Biocomplexes

**DOI:** 10.3390/molecules30030595

**Published:** 2025-01-28

**Authors:** Ilona E. Kłosowska-Chomiczewska, Gabriela Burakowska, Paulina Żmuda-Trzebiatowska, Aleksandra Soukup, Iwona Rok-Czapiewska, Elżbieta Hallmann, Tetiana Pokynbroda, Olena Karpenko, Krystyna Mędrzycka, Adam Macierzanka

**Affiliations:** 1 Chemical Faculty, Gdańsk University of Technology, G.Narutowicza 11/12, 80-233 Gdańsk, Poland; 2 Department of Physical Chemistry of Fossil Fuels Institute of Physical-Organic Chemistry and Coal Chemistry named after L. M. Lytvynenko, National Academy of Sciences of Ukraine, 3a Naukova Str., 79060 Lviv, Ukraine

**Keywords:** rhamnolipid biocomplex, alginate, biosurfactant purity, HIPE, emulsion

## Abstract

A novel, cost-effective, partially purified biosurfactant in the form of a rhamnolipid biocomplex (RLBC) was investigated for its emulsifying properties. The RLBC was obtained through the cultivation of *Pseudomonas* sp. SP-17 on glycerol, followed by acidic precipitation, without the use of organic solvents for isolation or purification. Composed of rhamnolipids (RLs) and the exopolysaccharide alginate, RLBC exhibited emulsifying properties towards rapeseed oil comparable to those of purified RLs at concentrations as low as 0.15% (*w*/*w*), sufficient for the effective stabilisation of *oil-in-water* (*o*/*w*) high internal phase emulsions (HIPEs, 80% oil). Dynamic light scattering analysis revealed similar droplet sizes (9.54 ± 0.96 µm for RLBC vs. 8.93 ± 0.58 µm for RLs), while multiple light scattering confirmed high emulsion stability over 120 days. The emulsions displayed shear-thinning behaviour, with yield stresses of approximately 11.5 Pa and 7.7 Pa for systems prepared with RLBC and RLs, respectively, after seven days of pre-storage. Although increasing the RLBC concentration from 0.15% to 1% (*w*/*w*) slightly improved the degree of emulsion dispersion, it did not substantially impact the long-term stability observed at the lowest concentration. Biodegradation tests demonstrated that the RLBC preparations are environmentally friendly alternatives to synthetic surfactants, achieving 60% biodegradation within 2.5 days and complete biodegradation within 14 days, which outperformed synthetic emulsifiers. The RLBC offers both environmental and economic advantages over purified RLs, including reduced production costs and the elimination of organic solvents. Our findings highlight the potential of RLBC for stabilising HIPEs in applications requiring sustainable and biodegradable formulations, such as cosmetics, lubricants, and industrial fluids widely manufactured and utilised today.

## 1. Introduction

Natural surfactants (biosurfactants) are a promising alternative for petroleum-based synthetic surfactants. They have been proven to be less toxic, easily biodegradable, active in wide ranges of pH and salinity [1], and mild to skin [2,3,4]. This has resulted in wide appreciation of biosurfactants, especially for their emulsifying properties, in a broad spectrum of applications, from oil tank cleaning [5,6], oil spill removal [6,7,8], and oil [6,9] and bitumen recovery [6,10] to the stabilisation of metalworking fluids [11,12] and cosmetic products [13,14,15]. Despite scientific efforts, biosurfactant production and application for industrial purposes is still limited, especially due to the high costs of feedstock and the isolation and purification of target products [16,17]. Therefore, the persistent problem is not only finding effective producers of biosurfactants, i.e., microorganisms, but also how to optimise the biosynthesis and isolation of these natural surfactants.

Different attempts have been made to reduce the cost of biosurfactant biosynthesis. One of the methods is to increase the efficiency of the production by the application of so-called overproducers—genetically engineered microorganism species with a high efficiency of biosurfactant production [8], which can increase rhamnolipid (RL) production up to 10-fold compared to the parental species [18]. Another method uses the application of waste materials as a carbon source for microorganisms. It is not only an important way of disposing of waste, but it also allows the reduction of the cost of culture medium for biosurfactant production, which has been estimated to amount to 10–30% of the total production cost [1]. Thus, hydrophilic waste products such as distillery and curd whey wastes [12], leachates from potato process effluent [19], cassava wastewater [20], molasses [21], or soapstocks and post-refining fatty acids [22,23,24] have all been successfully used for biosurfactant production. Hydrophobic waste substrates such as waste sunflower and olive oils [25], waste soya oil [24], or waste animal fats [24,26] have also been used. Additionally, the application of mixtures of both, hydrophobic and hydrophilic carbon sources, has been found to offer higher production efficiencies [27,28,29]. The exact impact of culture medium composition (e.g., carbon and nitrogen sources), as well as the parameters of biosynthesis (e.g., temperature, pH, and cultivation time) on the final composition and properties of the biosurfactant has been previously reported [30]. A new, prospective direction in biosurfactant manufacturing is the production of complexes of microbial surfactants with other metabolites, synthesised simultaneously while culturing the producer strain. To date, there are only limited data available in the scientific literature on the utilisation of such products [31,32,33,34], even though they either match or even surpass individual surfactants in physicochemical characteristics such as surface activity and micellization [32,34] and emulsification [34]. What is more, the complexes have revealed substantial antiviral activity [34] and anticorrosive properties at relatively low concentrations [33]. Importantly, the production of such complexes does not require extensive purification compared to when individual biosurfactants are obtained [32]. This offers a reduction in the consumption of organic solvents, which can save 60–80% of the total cost of production [35,36]. To our knowledge, the scientific literature about the stability and structure of emulsions formed in the presence of biosurfactant complexes is very scarce, and the available data do not offer solutions that can be considered economically viable [37].

Considering all the above, the purpose of our research was to compare the emulsifying properties of an RL biocomplex with alginate (RLBC), produced by *Pseudomonas* sp. PS-17, against pure RLs. The comparison served to guide potential applications of RLBC-stabilised emulsions as cosmetic formulations or metalworking fluids (MWFs). Therefore, special focus has been placed on the stability, structure, and rheology of emulsions.

## 2. Results and Discussion

### 2.1. Rhamnolipid Biocomplex Synthesis

The RLBC was produced according to a procedure that allowed for the omission of extraction and purification with organic solvents, which are typical for obtaining purified RLs [38,39,40,41,42]. In brief, the RLBC was produced by *Pseudomonas* sp. PS-17 using glycerol as the carbon source. Further, the complex was isolated from cell-free culture broth by acidic precipitation, and then re-precipitated for additional purification. The RLBC represented the third class of purity of biosurfactants [32] and was composed of the following (*w*/*w*): diRL 72.30 ± 0.45%, monoRL 7.25 ± 0.05%, acidic polysaccharide alginate 20.50 ± 0.50%, and traces of proteins [32].

### 2.2. Interfacial Properties

**Figure 1:**
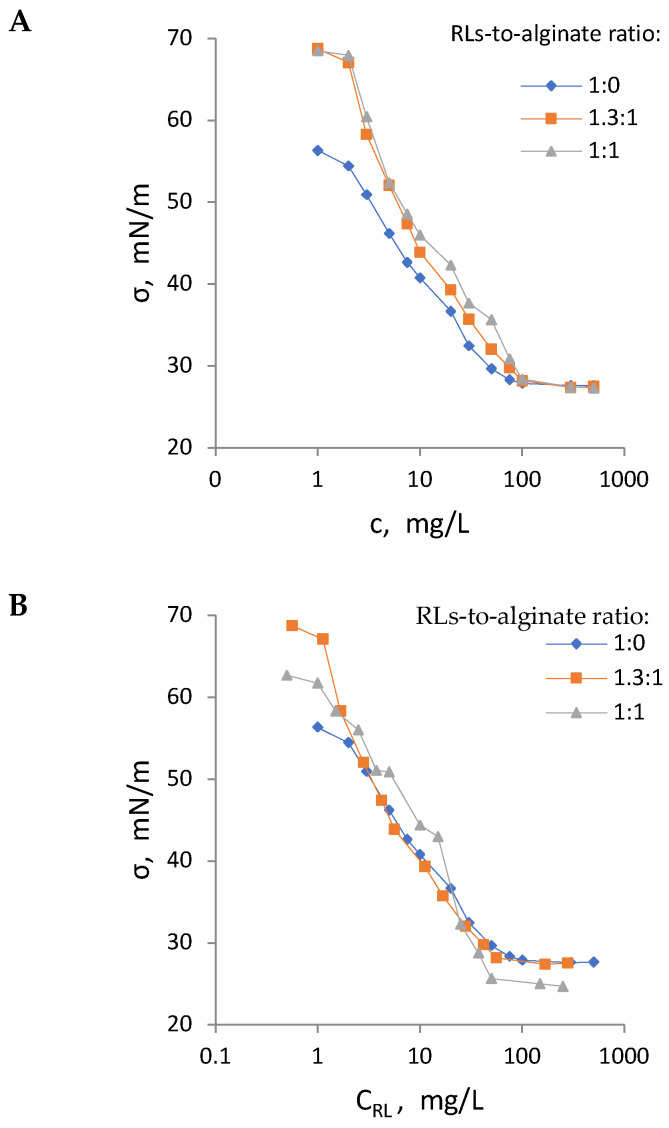
Surface tension isotherms for various RL preparations: RLs alone and their mixtures with alginate at 1.3:1 and 1:1 *w*/*w* ratios (model RLBCs). Isotherms are plotted against the concentration of the RL preparation as a whole (**A**) or recalculated based solely on the RL content within the RL preparation (**B**).

Surface tension measurements of model RLBCs (i.e., mixtures of RLs and alginate) were conducted to analyse the influence of alginate on the interfacial properties of RLs. RLs alone exhibited significantly greater surface activity compared to model RLBCs, resulting in lower surface tension at corresponding RL preparation concentrations (Figure 1A). Since RLs are surface-active and alginate is not considered to possess such properties, the data were replotted against the RL concentration in all analysed systems (Figure 1B). After replotting, the surface tension isotherms largely overlapped at RL concentrations above 3 mg/L, indicating that alginate does not affect the ability of RLs to reduce surface tension but decreases its relative contribution when applied in the form of model RLBCs.

Interfacial measurements were also used to determine the critical micelle concentration (CMC) of the RL preparations. With increasing alginate content, the CMC of the RL preparations rose, increasing from 72.4 mg/L for pure RLs to 87.6 mg/L at a 1:1 *w*/*w* RL-to-alginate ratio (Table 1). However, when the CMC values were recalculated based solely on the RL content, a decrease in the CMC was observed with increasing alginate share, dropping from 72.4 mg/L for pure RLs to 48.0 mg/L at a 1:1 *w*/*w* RL-to-alginate ratio (Table 1). These findings indicate that alginate might have promoted the formation of RL micelles in aqueous solutions.

### 2.3. Emulsifying Properties

The emulsifying properties of RLBC and RLs were examined using an MLS stability analysis of emulsions formed in their presence, with particular emphasis on backscattering (BS). In both cases, the produced emulsions were of the *o*/*w* type, which is typical for RLs [43] due to their high HLB value [44].

**Figure 2:**
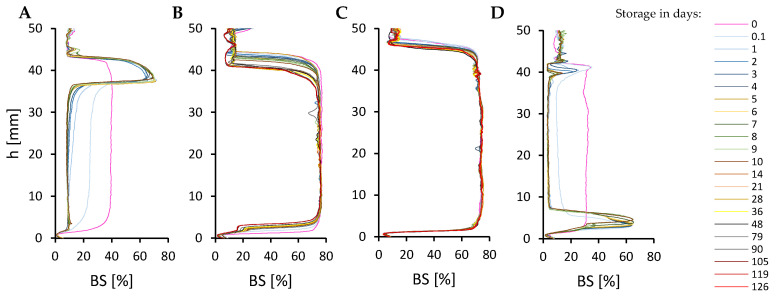
Backscattering (BS) analysis of emulsions stabilised by 0.15% (*w*/*w*) of RLBC and containing 10% (**A**), 70% (**B**), 80% (**C**), or 90% (**D**) of oil phase. Visible droplet migration instability (such as creaming) of emulsions containing up to 70% oil phase (e.g., BS peak formation in the uppermost location of the sample shown in (**A**) and phase separation in the emulsion with 90% oil (BS decrease over time and accumulation of remaining emulsion in the lower part of the sample shown in (**D**) can be seen)).

**Figure 3:**
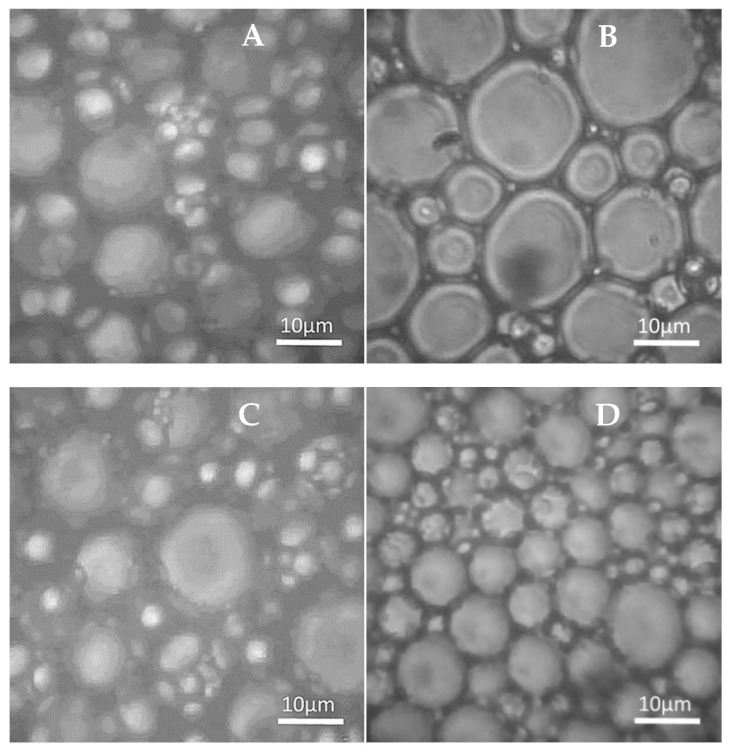
Microscopic structure of HIPEs (80% oil) stabilised with 0.15% (*w*/*w*) of purified RLs (**A**) or the RLBC biosurfactant at different concentrations (*w*/*w*): 0.15% (**B**), 0.25% (**C**), and 1.0% (**D**).

The minimum RLBC concentration required to form stable emulsions was experimentally determined to be 0.15% (*w*/*w*), with an optimal oil-to-water ratio of 80:20. Emulsions containing 10–70% oil phase were unstable, exhibiting creaming (Figure 2A,B). The extent of creaming decreased as the oil content in the emulsions increased, suggesting that droplet migration could be mitigated by their tight packing. In emulsions containing 90% oil, immediate phase separation occurred upon preparation (Figure 2D). However, emulsions containing 80% oil exhibited high and time-stable BS values, demonstrating the potential of RLBC to stabilise high internal phase emulsions (HIPEs). The closely packed oil droplets in these emulsions (Figure 3B) likely limited droplet migration due to steric hindrance.

The pKa of RLs is 5.6 [45], and the stability of the emulsions at the examined conditions (pH 9) was likely influenced by RL ionisation, as well as charge screening by Na⁺ ions [43]. Ionised RLs are known to form more elastic monolayers compared to their neutralised forms [46].

**Figure 4:**
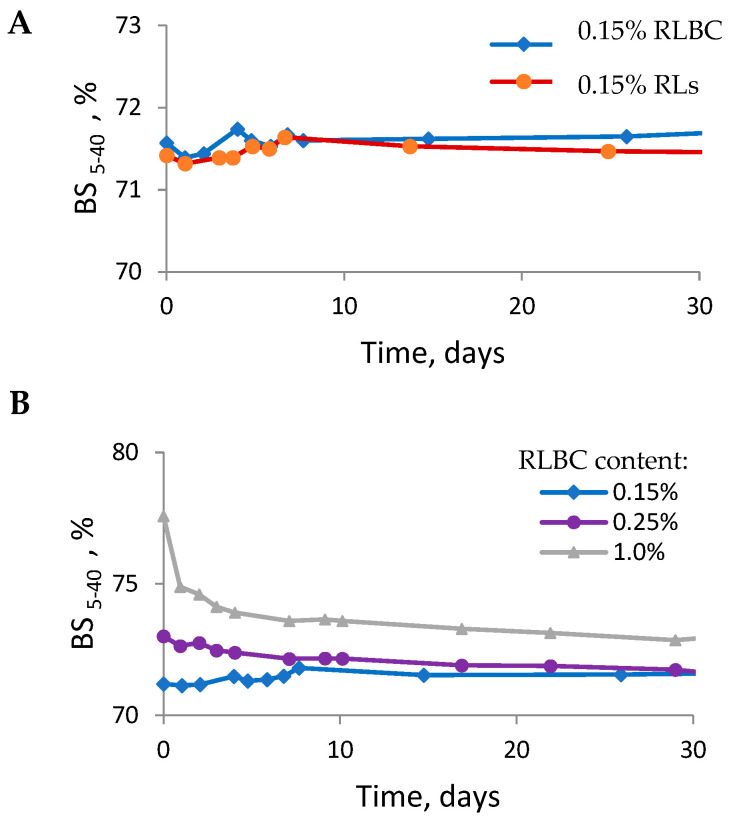
The time-dependent evolution of mean backscattering (BS_5-40_) for emulsions (80% oil phase) stabilised by 0.15% *w*/*w* of RLBC or purified RLs (**A**), or by the RLBC at different concentrations (**B**).

Emulsions stabilised with pure RLs were also prepared, and their BS_5–40_ profiles closely resembled those of emulsions stabilised with RLBC. Both RLBC- and RL-stabilised HIPEs (80% oil) exhibited very similar storage-dependent mean BS_5–40_ profiles (Figure 4A). The mean BS_5–40_ values were high (approximately 72%) and remained almost unchanged over the 30-day analysis period, indicating a high degree of dispersion and a stable microstructure of emulsions prepared with as little as 0.15% emulsifier.

HIPEs (80% oil) stabilised with higher RLBC concentrations (0.25% and 1.0%) were also prepared, and all the emulsions obtained were stable. As the RLBC content increased, the mean BS_5–40_ values were higher relative to those with 0.15% RLBC, but also showed greater variation over time (Figure 4B). This suggests that higher RLBC concentrations result in producing more dispersed systems, but also require more time for the final structure of emulsion to form—up to 10 days for systems stabilised with 1.0% RLBC.

Under the conditions used for emulsion formation (pH ca. 9), both RLs (pKa 5.6; [45]) and alginate (composed of mannuronic acid and guluronic acid units with pKa values of 3.38 and 3.65, respectively; [47]) were negatively charged. This charge might have resulted in intermolecular repulsion between RLs and alginate, as well as in intramolecular repulsion among the ionised carboxylic groups within alginate molecules. The latter might have induced steric hindrance, delaying RL adsorption at the interface to some extent. At high pH, the increased intramolecular repulsion leads to more expanded alginate structures [48]. As the RLBC concentration increased, the magnitude of repulsion likely intensified. This might have necessitated more time for the system to establish its final structure. Although increasing the RLBC concentration beyond 0.15% might have slightly enhanced the degree of emulsion dispersion, as described above, it is important to emphasise that this low emulsifier concentration was sufficient to maintain the stability of the analysed HIPEs during storage. It is also important to note that the presence of alginate in RLBC does not appear to substantially impact the emulsifying properties of RLs or the stability of the produced emulsions (Figure 4A). This could be attributed to the limited interference of alginate with the surface activity of RLs (Figure 1).

### 2.4. Droplet Size

**Figure 5:**
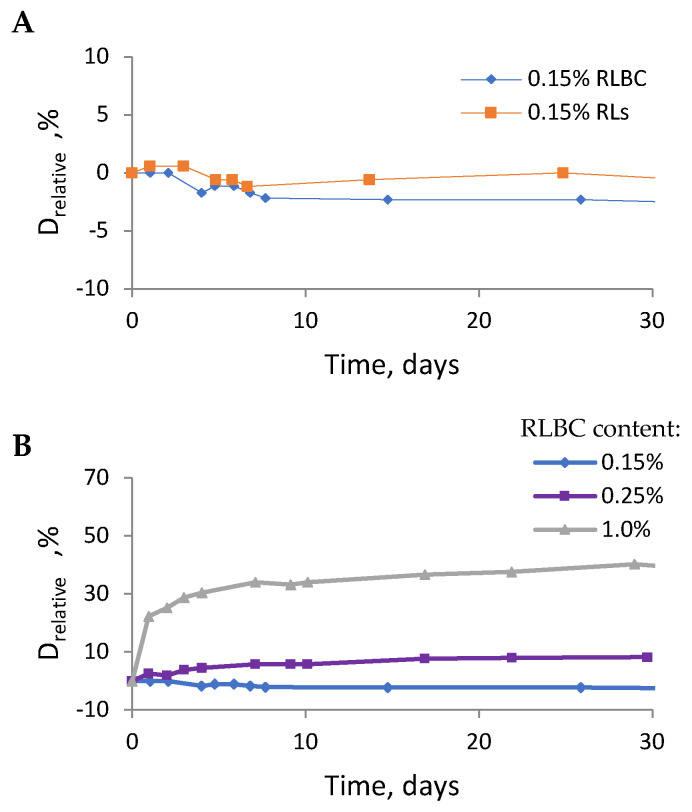
The relative change in oil droplet diameter for emulsions (80% oil phase) stabilised by 0.15% *w*/*w* RLBC or purified RLs (**A**), or by RLBC at different concentrations (**B**).

The average oil droplet size measured by DLS was 9.54 ± 0.96 μm for the 0.15% RLBC-stabilised emulsion, compared to 8.93 ± 0.58 μm for the emulsion stabilised with pure RLs. Both emulsions were relatively monodisperse, with PDI values of 0.339 ± 0.121 and 0.229 ± 0.084 for RLBC and RLs, respectively. Time-dependent relative changes in oil droplet diameter were calculated using MLS analysis (Figure 5). Consistent with the DLS results, the MLS-measured droplet size was similar for emulsions stabilised with either biosurfactant (i.e., RLBC or RLs) at 0.15% *w*/*w* (Figure 5A). Moreover, the MLS-based droplet size did not vary significantly over time, whereas notable changes in relative diameter were observed in emulsions prepared with higher RLBC concentrations (Figure 5B). The final droplet size of emulsions stabilised with 0.15% RL preparations was established within the first few days after preparation, with higher biosurfactant concentrations requiring more time for the emulsion’s final structure to develop. For example, in emulsions with 1.0% RLBC, the final oil droplet size required approximately 10 days to develop.

Microscopic analysis (Figure 3) provided insights about the morphology of the emulsions. Micrographs confirmed that the size and structure of systems stabilised with RLBC and RLs were similar (Figure 3A,B). Both emulsions were moderately monodispersed, with emulsion droplets ranging from ca. 5–15 µm, in accordance with the aforementioned average oil droplet size given by DLS (ca. 9–9.5 µm). The closely packed oil droplets exhibited deformation, a characteristic feature of HIPEs containing over 74% internal phase. Such deformations are consistent with rhomboidal dodecahedral packing [49].

### 2.5. Rheological Properties

**Figure 6:**
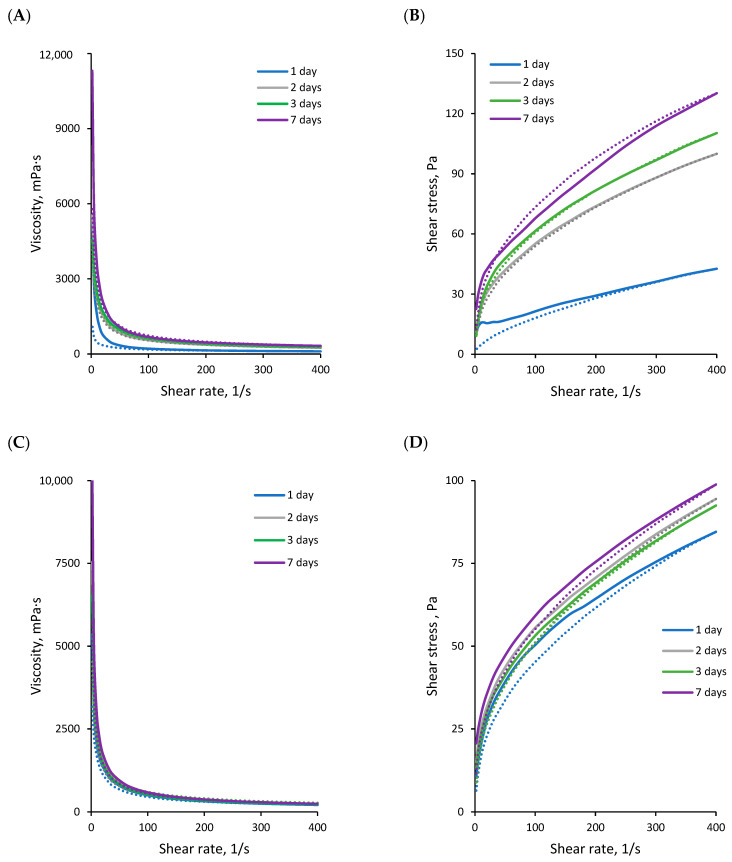
Storage time-dependent rheological properties of rapeseed oil emulsions (80%) stabilised with 0.15% RLBC (**A**,**B**) or purified RLs (**C**,**D**): viscosity (**A**,**C**) and flow curves (**B**,**D**) at ascending (solid lines) and descending (dashed lines) shear rates.

Specific rheological properties are of critical importance for emulsions used in various applications, e.g., as cosmetics or metalworking fluids (MWFs), as such products are subjected to shear during application. To evaluate the behaviour of the studied emulsions under dynamic conditions, their rheological properties were examined across a wide range of shear rates. The viscosity (Figure 6A,C) and flow curves (Figure 6B,D) of emulsions stabilised by either RLBC or RLs demonstrated characteristics typical of non-Newtonian pseudoplastic shear-thinning fluids, which is expected for emulsions containing over 60% dispersed phase [50], and can enhance, e.g., the spreadability of cosmetic emulsions or MWFs, and improves convection when used as cooling agents [51].

The rheological properties of the emulsions evolved during storage (Figure 6). This was particularly evident in the RLBC-stabilised emulsion. After two days of storage, the emulsion viscosity increased relative to the freshly prepared emulsion (Figure 6A), and it became more resistant to shear, as shown by its ability to recover viscosity in the experiment involving a shear cycle (Figure 6B). These time-dependent changes in rheological behaviour were consistent with MLS measurements, where fluctuations in BS_5–40_ were more pronounced during the first days after preparation for RLBC-stabilised emulsions compared to those stabilised with pure RLs (Figure 4A). This effect was likely due to the presence of alginate in the RLBC. Alginates have been shown to influence the rheological properties of *o*/*w* emulsions. For instance, increasing exopolysaccharide concentrations from 0.2% to 0.5% can enhance the storage modulus, with the effect becoming concentration-independent only for alginates with molecular weights of 76–120 kDa [52]. In *o*/*w* nanoemulsions, increased concentrations of alginate have been found to result in higher viscosity and more pronounced pseudoplasticity [53]. However, in our present study, the final viscosity curves (after 7 days of storage) of the RLBC-stabilised emulsions were similar to those of emulsions stabilised with pure RLs. This similarity is likely due to the low alginate content in the system (only 0.15% *w*/*w* RLBC was used, with alginate constituting approximately 20–21% of the RLBC, resulting in the final concentration of ca. 0.03% alginate in the system). Also, alginate alone exhibited poor emulsion stabilisation properties (Figure S1), as it is not considered surface-active.

The delayed development of the final emulsion structure can be attributed to two parallel phenomena. First, the lower concentration of surface-active molecules (RLs) in RLBC-stabilised emulsions compared to those stabilised with pure RLs—since both emulsions were prepared with the same 0.15% *w*/*w* stabiliser concentration in the aqueous phase—resulted in diffusion likely being the predominant factor affecting surfactant adsorption at the interface, leading to slower development of equilibrium [54]. Second, steric and electrostatic repulsion likely contributed to the delay. Under the aqueous-phase conditions (pH~9), both RLs (pKa 5.6; [45]) and alginate (composed of mannuronic and guluronic acid units with pKa values of 3.38 and 3.65, respectively; [47]) were negatively charged. This negative charge likely caused electrostatic repulsion between RLs and alginate, which might have hindered the migration of RL molecules towards the *o*/*w* interface. Consequently, the system might have reached equilibrium slower than in emulsions stabilised with RLs alone.

After reaching the final emulsion structure (7 days), the viscosity and flow curves for emulsions with RLBC or RLs were essentially similar (Figure 6). As demonstrated previously [50], this could be attributed to the comparable oil droplet size, which can significantly influence emulsion viscosity at high internal phase volumes. The rheological behaviour of the emulsions was best described by the Herschel–Bulkley mathematical model [55,56], expressed by the following equation:

τ = τ^o^ + kD^n^ (1)

where *τ* is the shear stress, τ^0^ is the yield stress, k is the consistency index, n is the flow index, and D is the shear rate.

The systems exhibited an initial resistance to flow, characteristic of HIPEs [57], which was more pronounced in the RLBC-stabilised emulsion. The yield stress values (τ^0^) were higher for the RLBC-stabilised emulsions than for systems stabilised solely with RLs (11.5 Pa vs. 7.7 Pa, respectively; Table 2). The effect was likely due to the presence of alginate in the RLBC. Ching et al. [58] reported that increasing the alginate concentration consistently raised the yield stress of emulsion-filled alginate microgel suspensions across all examined oil volume fractions, as confirmed by Herschel–Bulkley modelling and shear sweep analysis. In turn, the rheological behaviour of *o*/*w* emulsions stabilised by mixtures of proteins and alginate, described by the Power law model, has shown that increasing the concentration of alginate decreased the flow index (n) and increased the consistency index (k) of analysed systems [59], contrasting with our present results (Table 2). This highlights the role of alginate in providing structural integrity and resistance to the flow of the systems, which might depend on the accompanying surface-active molecules (e.g., surfactants and proteins).

### 2.6. Biodegradation Potential

The significance of emulsion biodegradability is critical, particularly in the context of cosmetics, where residues from personal care products ultimately reach wastewater treatment plants. If not adequately degraded in biological reactors, these residues can enter the environment. This concern is equally relevant for emulsions used as metalworking fluids (MWFs), with approximately 210,000 tons requiring disposal annually in Europe alone [12]. Therefore, it is essential to design emulsions using readily biodegradable substances to minimise their environmental impact. Although biosurfactants are widely recognised for their biodegradability, RLBC has not been examined in this context. Since purification of RLBC with organic solvents was excluded, we chose to investigate its biodegradation potential and compare it to its individual components—purified RLs and alginate. The potential was measured at controlled conditions, provided by OECD guidelines [60].

**Figure 7:**
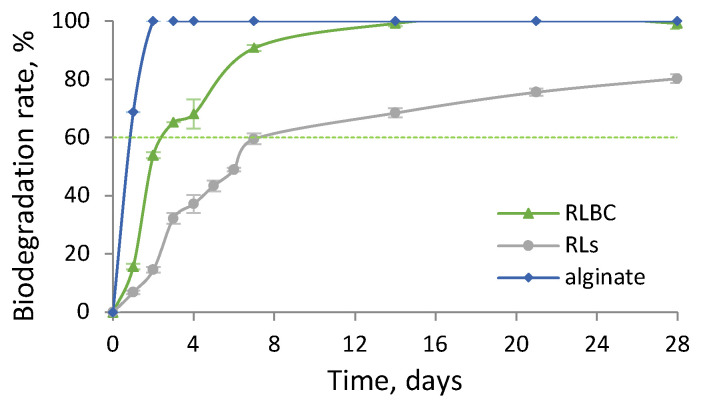
Time-dependent biodegradation rate for RLBC, RLs, and alginate.

The biodegradation curve for RLBC was intermediate compared to those of its individual components, RLs and alginate (Figure 7). All three substances were classified as readily biodegradable, as their biodegradation rates exceeded 60% within less than 10 days of the 28-day biodegradability test [60]. Alginate exhibited the fastest biodegradation, reaching over 60% within less than one day, indicating excellent biodegradability. For RLBC and RLs, this threshold was reached after approximately 2.5 days and 7 days, respectively. The final biodegradation rates after 28 days were 100%, 80%, and 100% for RLBC, RLs, and alginate, respectively (Figure 7).

Reaching 60% biodegradation in approximately 2.5 days (Figure 7), the RLBC represents an eco-friendly alternative to synthetic surfactants. In comparison, the biodegradation of other emulsifiers commonly used for stabilising lubricating and multi-purpose emulsions, such as ethoxylated lauryl alcohols (nonionic, C_n_EO_m_) [61,62], cationic CTAB, or anionic glycolic acid ethoxylate oleyl ether (Oleth 10) [61], was significantly slower.

For ethoxylated alcohols, reaching 60% biodegradation required 8 days for C_12−15_EO_7_, 15 days for C_12−15_EO_9_ [63], and 7–20 days for C_10−12_EO_5_, depending on the concentration (250–1000 g/L) [64]. In the case of C_18_EO_10_, a long lag phase of over 8 days was observed during respirometric tests in liquid, and it was found to be non-biodegradable in soil respirometric tests [65].

For cationic CTAB, only 20% biodegradation was achieved within 14 days in a closed-bottle test [66], while no biodegradation data were available for Oleth 10.

## 3. Materials and Methods

### 3.1. Materials

The RLBC was synthesised using Pseudomonas sp. PS-17, provided by the Department of Physical Chemistry of Fossil Fuels at the Institute of Physical-Organic Chemistry and Coal Chemistry (National Academy of Sciences of Ukraine). The strain was cultivated in a liquid nutrient medium containing the following (g/L): NaNO_3_—4.0; K_2_HPO_4_∙3H_2_O—2.0; KH_2_PO_4_—1.2; MgSO_4_∙7H_2_O—0.5; sodium citrate—4.0 g; distilled water up to 1 L; and 4% (wt.) of glycerol as the carbon and energy source. The medium was inoculated with a 1-day-old inoculum of *Pseudomonas* sp. PS-17 (5% of medium volume) [67]. After 5 days of cultivation, the biomass was separated by centrifugation (6000 rpm, 20 min), and the obtained supernatant was acidified to pH 3–4, followed by cooling (4 °C, 12 h). The resulting biosurfactant precipitate (RLBC) was separated through centrifugation (8000 rpm, 20 min) [68]. The obtained RLBC was freeze-dried (CHRIST Alpha 2-4 LSC, Osterode am Harz, Germany) prior to use. Purified RLs were obtained from Jeneil Biotech Inc. (Saukville, WI, USA) as 25% the aqueous solution of dirhamnolipid (diRL) and monorhamnolipid (monoRL) in a 0.97:1 ratio (JBR 425). Alginic acid (A2033) was purchased from Sigma Aldrich. Rapeseed oil (VOG, Skierniewice, Poland) was chosen as the oil phase, since it is the most commonly produced oil in Poland [69,70], a valuable cosmetic ingredient and a source of polyunsaturated fatty acids for cosmetic purposes [71,72,73], and an easily biodegradable alternative for mineral oils used in MWFs [11,12,74]. It was purchased at a local market. The composition of the oil FAs was determined by GC as follows, 59.7 ± 0.3% C18:1, 19.2 ± 0.4% C18:2, 9.7 ± 0.2% C18:3, 4.5 ± 0.1% C16:0, 1.6 ± 0.1% C18:0, 1.21 ± 0.05% C20:1, 0.58 ± 0.04% C20:0, 0.14 ± 0.02% C22:0, 0.03 ± 0.01% C14:0, 0.02 ± 0.01% C12:0, and was typical for rapeseed oils [75].

### 3.2. Interfacial Tension Measurements

Interfacial tension σ of pure RLs and model RLBCs, composed of RLs and alginate at 1:1 and 1.3:1 *w*/*w* ratios, was measured with the pendant drop technique using a DSA 10 analyser (Krüss GmbH, Hamburg, Germany). The method relies on the analysis of the equilibrium shape of a drop suspended in air. The drop contour was traced using image analysis software, and the Young–Laplace Equation (2) was fitted to the drop profile to compute σ:

(2) $$\Delta P=\sigma \;(\frac{1}{R_{1}}+\frac{1}{R_{2}})$$

where ΔP is the pressure difference across the interface in Pa, R_1_ and R_2_ are the radii of curvature in m, and σ is the surface tension in mN/m. Measurements were performed at a controlled temperature of 25 °C to ensure consistency. Biosurfactant concentrations ranged from 0.001 g/L to 0.5 g/L. For each concentration of biosurfactant solution, measurements were repeated for three separate drops to ensure precision and reproducibility. The setup was validated using water as a reference system. The critical micelle concentration (CMC) was determined by plotting σ against the logarithm of surfactant concentration. The CMC was identified as the concentration at which two linear regions (below and above CMC) intersect.

### 3.3. Preparation of Emulsions

The biosurfactants were dissolved in ultra-pure water (κ = 0.07 μS, HLP5s, Hydrolab, Straszyn, Poland) at concentrations of 0.15%, 0.25%, or 1.0% *w*/*w*, and the pH of the solution was set to 9 with 1M NaOH to increase their solubility. Subsequently, the oil phase was added to surfactant solution and the mixture was homogenised (8000 rpm, 10 min; Yellow Line basic DP25, IKA, Warsaw, Poland). The oil-to-water phase ratios ranging from 10/90 to 90/10 (*w*/*w*) were examined. Additionally, the emulsions stabilised with 0.15% alginate alone were also examined.

### 3.4. Emulsion Stability Measurements with Multiple Light Scattering

The stability of the emulsions was determined with the multiple light scattering technique (MLS) using a Turbiscan tLab Expert analyser (Formulaction, Toulouse, France). Freshly prepared emulsions (ca. 25 mL) were placed into a flat-bottomed cylindrical glass cell and stored at 23 ± 1 °C. The samples were scanned by two synchronous optical sensors detecting the intensity of light transmitted through and backscattered by the sample (180° and 45° from the incident laser light, respectively). The optoelectronic head acquired transmission (T) and backscattering (BS) data every 40 μm while scanning along the entire height of the cell (55 mm). The light source was an electro-luminescent diode in the near-infrared region (λ_air_ = 880 nm).

The specifics of the multiple light scattering (MLS) technique employed with the Turbiscan tLab Expert analyser are described in detail in a previous study [76]. The relationship between the intensity of backscattered light and the photon transport length (λ*) through the dispersed system was utilised to determine relative changes in droplet size within emulsions. The equations governing these relationships are provided below:

(3) $$BS\approx \frac{1-B}{3}\sqrt{\frac{dh}{\lambda^{*}}}$$

(4) $$\lambda^{*}=\frac{2d}{3\phi \left(1-g\right)Q_{s}}$$

where B ≈ 0.2 is the boundary reflection factor in cylindrical geometry and *h* is the detector height. The optical parameters Q_s_ and g are given by Mie theory. The relative change in droplet size D_rel_ was calculated as follows:

(5) $$D_{\mathrm{rel}}=\frac{d-d_{0}}{d_{0}}\cdot 100\%$$

where *d_0_* is the droplet diameter just after emulsion preparation and *d* is droplet diameter at a certain time. The mean BS values (BS_5-40_) for stable emulsions (no creaming or coalescence observed) were calculated at a 5–40 mm height of the glass cell containing the emulsion.

### 3.5. Emulsion Droplet Size Measurements

The average diameter of dispersed-phase droplets was measured with the dynamic light scattering (DLS) technique using a Zetasizer Nano ZS (Malvern Instruments Ltd., Malvern, UK). One drop of emulsion (ca. 3 μL) was diluted about 6000 times with ultra-pure water to avoid multiple particle scattering effects [77]. All measurements were carried out in triplicate at 25 ± 0.1 °C. Each measurement consisted of 4 sets, with 10 runs each.

### 3.6. Microscopic Analysis

The morphology of emulsions was investigated using a Biolar 2308 microscope (PZO, Warszawa, Poland) equipped with achromatic objectives and a VF0260 digital camera (Creative, Singapore). A thin layer of the emulsion was placed on the microscope slide and covered with a cover glass. The sample (ca. 50–100 µL) was collected with a cut-end pipette tip (200 µL, ϕ ca. 2 mm) and from the bulk layer of the emulsion.

### 3.7. Rheological Measurements

Emulsions (ca. 2 mL) stabilised with 0.15% of the RLBC or RLs were analysed using a DV2THA viscometer (Brookfield, Middleboro, MA, USA) with cone-plate geometry (24 mm diameter, 3° angle) at 25 ± 0.1 °C. Rheological measurements were conducted after 1, 2, 3, and 7 days of storage, with the shear rate ascending from 2 to 400 s^−1^ and then descending back to 2 s^−1^. Viscosity and shear stress were recorded continuously. Flow curves were analysed for hysteresis and modelled using various constitutive equations to characterise flow behaviour.

### 3.8. Biodegradation Potential Assesment

The susceptibility of RLBC and its main components, i.e., RLs and alginate, to biodegradation was assessed according to ISO 10707:2002 [78] and OECD 301D Closed Bottle Test recommendations [60], widely used for screening the biodegradation potential of various chemicals, including surfactants [79,80,81], antibiotics [82,83], ionic liquids [84], deep eutectic solvents [85], and polymers for pharmaceutical formulation [86]. Samples were prepared in accordance with the guidelines for poorly soluble substances (ISO 10634:2001) [87]. The test substance (4 mg/L) was homogenised with dilution water (15,000 rpm, 10 min; Yellow Line DI 25 basic homogenizer, IKA, Warsaw, Poland), bottled, and then kept in the dark at a constant temperature 20 ± 1 °C. Biodegradation (28 days) was carried out simultaneously for two independent series of solutions for each substance tested. The concentration of dissolved oxygen was investigated in the samples with the Winkler method [88]. Ethylene glycol was used as a reference substance. The dilution water was prepared according to the OECD guidelines [60] and ISO 10707:2002 [78], namely, 1 mL/L of each mineral salt, i.e., CaCl_2_, MgSO_4_∙7H_2_O, and FeCl_3_∙6H_2_O solutions and phosphate buffer pH 7.4, was added to distilled water, and then the solution was inoculated with supernatant liquid obtained from the nitrification chamber of Wschód Wastewater Treatment Plant (Gdańsk, Poland) and aerated for 48 h.

The degree of biodegradation was calculated using the following formula [60]:

(6) $${\%}_{biodegradation}=\frac{BOD}{TOD}\cdot 100\%$$

where BOD is the biochemical oxygen demand (mg O_2_/mg) calculated on the basis of dissolved oxygen concentration in samples before and after incubation, and TOD is the theoretical oxygen demand (mg O_2_/mg) calculated based on the chemical structure of the compounds (Table S1). Considering the C_c_H_h_Cl_cl_N_n_Na_na_O_o_P_p_S_s_ structural formula of the examined compound, the TOD is expressed as follows:

TOD = 16 × [2c + 1/2 (h − cl − 3n) + 3s + 5/2p + 1/2 na − o]/MW [mgO_2_/mg] (7)

where MW is the molecular weight of the examined compound (g/mol).

## 4. Conclusions

In this study, the emulsifying properties and biodegradability of a partially purified rhamnolipid/alginate biocomplex (RLBC), produced by *Pseudomonas* sp. PS-17, were characterised for the first time. The RLBC demonstrated emulsifying properties comparable to those of purified RLs and enabled the formation of stable high internal phase *o*/*w* emulsions (HIPEs) containing 80% rapeseed oil. The stability, average oil phase droplet size, and final rheological properties of the RLBC-stabilised emulsions were similar to those of RL-stabilised emulsions. However, the structuring of RLBC-stabilised emulsions required more time (approximately 7 days after preparation) compared to RL-stabilised emulsions. This delay was attributed to the lower RL content in the RLBC and electrostatic repulsion between RLs and alginate. Further research is necessary to investigate the specific contributions of the RLBC components to emulsion stabilisation and to determine the optimal concentration for manufacturing HIPEs.

The RLBC-stabilised HIPE systems may be of interest in designing cosmetics [89,90,91,92] and food products [93] and could also serve as lubricants. HIPEs have been shown to improve plate-out oil formation [94] and produce thicker films compared to dilute emulsions [95]. Using the RLBC instead of purified RLs to stabilise HIPEs can offer additional ecological and economic benefits, including the high biodegradation potential of the RLBC (surpassing that of purified RLs or other emulsifiers typically used for stabilising MWFs), solvent-free production, and a cost reduction. The market prices of highly purified rhamnolipids (RLs) vary substantially depending on the product form and packaging size. For instance, powder formulations containing 90% RLs are currently priced at USD 7–39 per gram for small packages (10–100 g) from suppliers such as Merck (Darmstadt, Germany) or at USD 105–135 per kilogram for bulk orders (25–100 kg) from AGAE Technologies (Corvallis, OR, USA). Commercially available solutions with 25% RL content are priced at USD 19 per litre (AGAE Technologies, Corvallis, OR, USA) or USD 110 per kilogram (Jeneil Biotech, Saukville, WI, USA). The pricing excludes delivery charges but includes unspecified company mark-ups. In comparison, the estimated production cost of biosurfactant formulations—including biosynthesis using industrial waste, isolation, extraction, transportation, and taxes—is approximately USD 0.02 per gram [96]. Considering that the RLBC production methodology reduces solvent purification, which accounts for 60–80% of total production costs [35,36], the final price of the RLBC might be potentially reduced to a fraction of the current market prices of purified RLs.

## Figures and Tables

**Table 1:** Micellization properties of aqueous solutions of purified RLs and their mixtures, with alginate at pH 9, determined using pendant drop shape analysis. The critical micelle concentration is presented for the whole mixture (CMC) and recalculated based on the RL content in the mixtures (CMC_RL_).

RL:Alginate Ratio (*w*/*w*)	Alginate Share in Mixture	cmc _RL_	cmc	σ at cmc
	*%*	mg/L	mg/L	mN/m
1:0	0	72.4	72.4	26.8
1.3:1	43	52.7	80.5	26.5
1:1	50	48.0	87.6	26.5

**Table 2:** Rheological parameters of biosurfactant-stabilised emulsions (after 7 days of storage) derived from the Herschel–Bulkley mathematical model.

Biosurfactant	Consistency Index k, mPa∙s	Flow Index n	Yield Stress τ^o^, Pa	Confidence of Fit, %
RLBC	6 359	0.488	11.50	99.1
RLs	6 803	0.431	7.72	99.0

## Data Availability

The original contributions presented in this study are included in the article/Supplementary Materials. Further inquiries can be directed to the corresponding author(s).

## Supplementary Materials

The following supporting information can be downloaded at https://www.mdpi.com/article/10.3390/molecules30030595/s1: Figure S1. Emulsifying properties of alginate: (A) emulsions with different amount of rapeseed oil ranging from 10 to 90%; (B) transmission (T) and backscattering (BS) profiles obtained with multiple light scattering technique for alginate stabilized emulsion containing 80% of rapeseed oil. Rapid changes of both T and BS in time indicate fast creaming of emulsions formed in presence of alginate; Table S1. Main assumptions regarding the composition of rhamnolipid biocomplex (RLBC), rhamnolipids (RLs) and alginate taken into consideration for theoretical oxygen demand (TOD) calculations.
