# Supplementary material for: High Internal Phase Oil-in-Water Emulsions Stabilised by Cost-Effective Rhamnolipid/Alginate Biocomplexes
Source: Molecules. 2025 Jan 28;30(3):595. doi: 10.3390/molecules30030595 (PMC11821110; doi:10.3390/molecules30030595)
Supplement: Supplementary file 1 [file molecules-30-00595-s001.zip › molecules-3414037-supplementary.pdf]

High internal phase o/w emulsions stabilised by cost-effective rhamnolipid/alginate biocomplexes

Ilona E. Kłosowska-Chomiczewska [1,*], Gabriela Burakowska [1], Paulina Żmuda-Trzebiatowska [1], Aleksandra Soukup [1], Iwona Rok-Czapiewska [1], Elżbieta Hallmann [1], Tetiana Pokynbroda [2], Olena Karpenko [2], Krystyna Mędrzycka [1] and Adam Macierzanka [1,*]

1 Chemical Faculty, Gdańsk University of Technology, G.Narutowicza 11/12, 80-233, Gdańsk, Poland
2 Department of Physical Chemistry of Fossil Fuels Institute of Physical-Organic Chemistry and Coal Chemistry named after L. M. Lytvynenko, National Academy of Sciences of Ukraine, 3a Naukova Str., 79060 Lviv, Ukraine
* Correspondence: ilochomi@pg.edu.pl (I.E.K.-C.); adam.macierzanka@pg.edu.pl (A.M.); Tel.: +00-48-58-347-11-51 (I.E.K.-C.); +00-48-58-347-29-27 (A.M.)

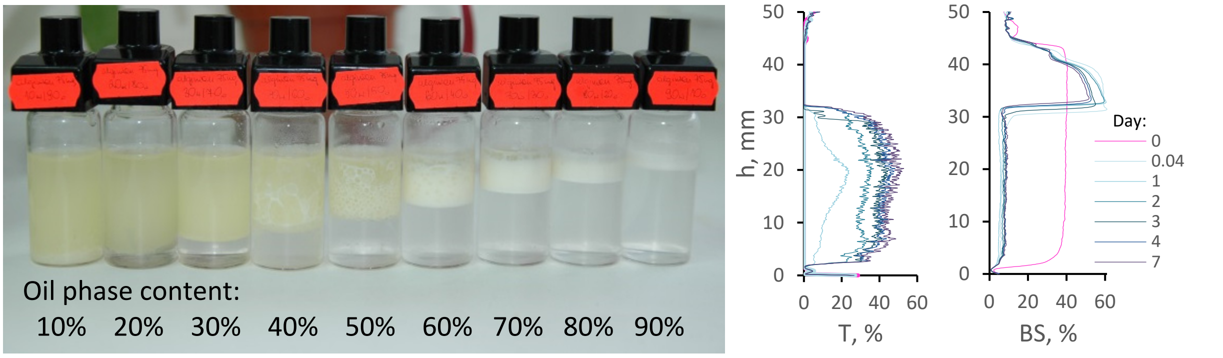

Figure S1. Emulsifying properties of alginate: A) emulsions with different amount of rapeseed oil ranging from 10 to 90%; B) transmission (T) and backscattering (BS) profiles obtained with multiple light scattering technique for alginate stabilized emulsion containing 80% of rapeseed oil. Rapid changes of both T and BS in time indicate fast creaming of emulsions formed in presence of alginate.

Table S1. Main assumptions regarding the composition of rhamnolipid biocomplex (RLBC), rhamnolipids (RLs) and alginate taken into consideration for theoretical oxygen demand (TOD) calculations.

**Substance**	**Assumption**	**Formula assumed**	**TOD** *mgO₂/mg*
RLBC	The composition of RBC was published elsewhere [1]	alginate: $(C_6H_8O_6)_n$ (n = 1989)<br>RRLL: $C_{32}H_{58}O_{13}$<br>RLL: $C_{26}H_{48}O_9$	**1.763**
RLs	RRLL:RLL = 0.97:1 (w/w)<br>(product data)	RRLL: $C_{32}H_{58}O_{13}$<br>RLL: $C_{26}H_{48}O_9$	**2.049**
alginate	mean MW = 350 000 g/mol [2]	$(C_6H_8O_6)_n$<br>where n = 1989	**0.909**

[1] I.E. Kłosowska-Chomiczewska, K. Mędrzycka, E. Hallmann, E. Karpenko, T. Pokynbroda, A. Macierzanka, C. Jungnickel, Rhamnolipid CMC prediction, Journal of Colloid and Interface Science, 488 (2017) 10-19.

[2] A. Shulga, E. Karpenko, S. Yeliseev, R. Vildamova-Martysyshyn, Pseudomonas sp. PS-17 strain is a producer of extracellular bio-surfactants and biopolymer, Ukrainian Patent, no. 71792 A,, 1996.
